# Designing a Pediatric Severe Sepsis Screening Tool

**DOI:** 10.3389/fped.2014.00056

**Published:** 2014-06-16

**Authors:** Robert J. Sepanski, Sandip A. Godambe, Christopher D. Mangum, Christine S. Bovat, Arno L. Zaritsky, Samir H. Shah

**Affiliations:** ^1^Department of Performance Improvement, Children’s Hospital of The King’s Daughters, Norfolk, VA, USA; ^2^Department of Pediatrics, Eastern Virginia Medical School, Norfolk, VA, USA; ^3^Department of Pediatrics, University of Tennessee Health Science Center, Memphis, TN, USA

**Keywords:** severe sepsis, screening tool, algorithm, emergency department, SIRS

## Abstract

We sought to create a screening tool with improved predictive value for pediatric severe sepsis (SS) and septic shock that can be incorporated into the electronic medical record and actively screen all patients arriving at a pediatric emergency department (ED). “Gold standard” SS cases were identified using a combination of coded discharge diagnosis and physician chart review from 7,402 children who visited a pediatric ED over 2 months. The tool’s identification of SS was initially based on International Consensus Conference on Pediatric Sepsis (ICCPS) parameters that were refined by an iterative, virtual process that allowed us to propose successive changes in sepsis detection parameters in order to optimize the tool’s predictive value based on receiver operating characteristics (ROC). Age-specific normal and abnormal values for heart rate (HR) and respiratory rate (RR) were empirically derived from 143,603 children seen in a second pediatric ED over 3 years. Univariate analyses were performed for each measure in the tool to assess its association with SS and to characterize it as an “early” or “late” indicator of SS. A split-sample was used to validate the final, optimized tool. The final tool incorporated age-specific thresholds for abnormal HR and RR and employed a linear temperature correction for each category. The final tool’s positive predictive value was 48.7%, a significant, nearly threefold improvement over the original ICCPS tool. False positive systemic inflammatory response syndrome identifications were nearly sixfold lower.

## Introduction

### Background

Pediatric severe sepsis is a serious condition with worldwide significance. Severe sepsis (SS) is defined as acute organ dysfunction (OD) in the presence of sepsis; the latter refers to the presence of a systemic infection, which can result from a bacterial, viral, or fungal source. SS is a leading cause of multiple organ failure and mortality across intensive care units (1). In the United States, there are an estimated 751,000 cases/year with an annual cost of $17 billion (1–5). Between 20,000 and 40,000 US children develop septic shock annually, and its incidence is rising (6, 7).

Despite basic and clinical research efforts, SS and septic shock mortality remain largely unchanged over the past 20+ years, ranging from 23 to 50% (8, 9). To improve SS-related mortality, several organizations published evidence-based guidelines for the management of SS and septic shock (8, 10, 11). These guidelines provide a comprehensive bundle of recommended therapies for clinicians that if effectively implemented, could improve patient outcomes and reduce death. These guidelines include several time-sensitive interventions, such as antibiotic administration and fluid resuscitation, emphasizing the importance of early recognition of shock and sepsis (12). Although a recent study questioned the utility of such “early goal-directed therapy” (EGDT) measures for adult septic shock cases, the importance of early detection and initiation of antibiotic therapy nevertheless remains unchallenged (13). Moreover, detection of SS in children is often more difficult at least in part because of their greater ability to compensate during early stages of septic shock (14).

### Importance

Presently, the diagnosis of SS (which will henceforth be understood to also include cases that progressed to septic shock) is highly dependent on the clinical acumen of the caregiver and thus potentially subject to error. Creating an effective screening tool for children is challenging because vital signs [i.e., heart rate (HR), respiratory rate (RR), and blood pressure] and some laboratory values are age-dependent. While effective SS screening tools have been created for adults (15) and a proposed set of consensus-derived guidelines for a pediatric SS screening tool was published by the International Consensus Conference on Pediatric Sepsis (ICCPS) (2, 16), a similar validated tool of high predictive value for children has yet to be developed. Moreover, our preliminary testing of a screening tool based on the ICCPS guidelines resulted in high numbers of false positive SS alerts that could lead to “trigger fatigue” in a pediatric emergency department (ED) setting.

### Goals of this investigation

Beginning with the recommended components of a SS screening tool and age-specific criteria for vital signs put forth by ICCPS, we empirically identified new vital sign thresholds and applied our tool refinement methodology to create an improved tool for detection of SS in terms of specificity, positive predictive value (PPV), and median time from patient arrival to SS detection. Although the tool was refined using retrospective patient data, our goal is to create an automated, real-time electronic version of the tool that will be incorporated into the hospital electronic medical record (EMR) and will actively screen all patients arriving at the pediatric ED.

## Materials and Methods

### Study design and setting

Our study refined and tested an electronic screening tool for pediatric SS initially based on the ICCPS criteria. The refinement process utilized a retrospective database containing demographic, episode of care, and clinical data for all pediatric patients who visited the ED of a large, metropolitan children’s hospital over a 2-month period. The collected data spanned the entire hospital encounter of each patient, regardless of whether this involved only an ED visit or continued as an observation or inpatient admission to the hospital.

Although based upon retrospective data, the screening tool evaluated the data for each case in chronological order in a manner that emulated the function of a real-time tool, allowing the tool to “fire” – indicating that the criteria for SS were met – at any time during the simulated episode of care for that patient. The tool’s determination of a case as “positive” or “negative” for SS was then compared with an independent “gold standard” evaluation based on physician chart review and coded discharge diagnoses.

An important component of our screening tool is the identification of abnormal values for HR and RR in patients arriving at the pediatric ED. Previous attempts to establish age-specific ranges of normal and abnormal HR and RR, such as those suggested by ICCPS, employed consensus values based on small numbers of healthy, resting children and may not be appropriate for children presenting to an ED. A recent study (17) suggested that empirically derived upper thresholds of normal HR and RR in pediatric inpatient hospital settings are considerably higher than these previously used consensus values. Similarly, our study included an empirical analysis of initial ED triage vital signs from over 140,000 children in order to derive age-specific values for normal and abnormal HR and RR in a pediatric ED setting. The resulting redefinition of age-specific abnormal vital sign values for pediatric ED patients was an essential precursor in the subsequent refinement process that sought to create a screening tool with substantially improved performance.

The evaluation and refinement of our pediatric SS screening tool utilized virtual plan-do-study-act (PDSA) cycles with the goal of optimizing the tool’s receiver operating curve (ROC) characteristics – sensitivity, specificity, PPV, and area under the curve (AUC) – while simultaneously attempting to minimize the time from patient arrival to detection of children identified as genuine cases of SS.

### Selection of participants

The primary study group consisted of all pediatric patients aged <18 years (*N* = 7,402) who presented to the ED of Le Bonheur Children’s Hospital (Memphis, TN, USA) during January and February, 2011. We refer to this group as the “Screening Tool Refinement Group.”

A second, independent group of all pediatric patients aged <18 years (*N* = 143,603), who presented to the ED of the Children’s Hospital of The King’s Daughters (Norfolk, VA, USA) between May, 2009 and September, 2012 and had electronically recorded measurements of HR, RR, and temperature taken at the time of triage, were selected in order to ascertain age-specific “normal” and “abnormal” values for HR and RR for pediatric patients in a hospital ED setting. We refer to this group as the “Vital Signs Standardization Group.” Various values for empirically derived normal and abnormal HR and RR from the Vital Signs Standardization group were then tested for their ROC performance using the patient data from the Screening Tool Refinement group in the ongoing tool refinement process.

The Screening Tool Refinement component of our study was approved by the Institutional Review Board (IRB) at Le Bonheur Children’s Hospital, and the Vital Signs Standardization component was approved by the IRB at Eastern Virginia Medical School (Norfolk, VA, USA).

### Methods and measurements

#### Screening tool refinement group

Specific data elements of the EMR over the course of each patient’s hospital encounter were obtained from the hospital’s Cerner database using the PowerInsight^®^ data extraction tool (Cerner Corporation, Kansas City, MO, USA). Data elements included the following: (1) patient characteristics and demographics; (2) episode of care information (admission and discharge dates and times for each hospital unit visited); (3) vital signs and clinical assessments; (4) details regarding the use of supplemental oxygen, mechanical ventilation, extracorporeal membrane oxygenation (ECMO), and emergency resuscitation events; (5) laboratory tests and results; (6) information on administered medications (asthma or seizure drugs, vasoactive agents, beta blockers, and clonidine); and (7) patient history as entered during ED triage.

**Figure 1:**
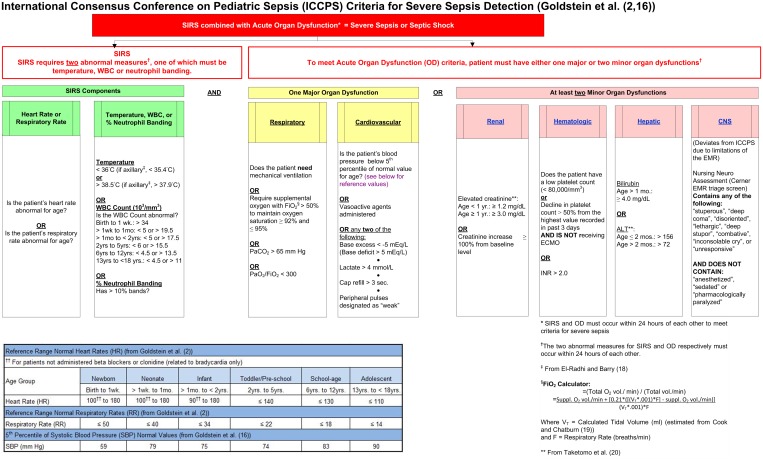
**Initial severe sepsis screening tool based on ICCPS (2) criteria**.

An electronic screening tool for pediatric SS was designed using SAS^®^ (v 9.3) (SAS Institute Inc., Cary, NC, USA) programing language. The tool algorithm, which determined if and when a positive firing occurred in each case, was based on the published ICCPS criteria, which were modified slightly to accommodate the availability of data from the patients’ EMR. The criteria employed in this initial version of the tool are summarized in Figure 1 (2, 16, 18–20).

To provide an independent assessment of the tool’s performance, systematic physician chart reviews – with the goal of identifying cases as positive or negative for SS – were performed on all cases in the Screening Tool Refinement group that met one of the following criteria: (1) a coded discharge diagnosis of one or more of: “severe sepsis” or “septic shock;” a disseminated bacterial, fungal, or viral infection; or a localized infection or related condition having the potential for progression to sepsis (see Table 1, which lists the specific ICD-9-CM coded diagnoses selected for chart review); or (2) a positive identification of SS (i.e., a “firing”) by the version of the tool being tested. A total of 480 cases met one or more of the above criteria and were selected for chart review. For each instance of chart review, the reviewing physician searched for evidence of SS, defined below as the presence of infection accompanied by systemic inflammatory response syndrome (SIRS) and OD, and was blinded as to the tool’s independent assessment of the case. For indeterminate cases, the final determination was made by joint physician review conducted by two physicians. The final result of this review process was the identification of “gold standard” SS cases that served as the standard for the assessment and refinement of the tool.

**Table 1:** **List of ICD-9-CM (21) coded diagnoses selected for chart review**.

**A. DISSEMINATED INFECTIONS**			
003.1	Salmonella septicemia	771.81	Septicemia (sepsis) of the newborn
018.xx	Miliary tuberculosis	771.83	Bacteremia of the newborn
020.2	Septicemia plague	785.52	Septic shock
022.3	Anthrax septicemia	790.7	Bacteremia
031.2	Disseminated mycobacteremia	790.8	Viremia, unspecified
036.2	Meningococcemia	995.90	Systemic inflammatory response syndrome (SIRS)
038.xx	Septicemia	
040.82	Toxic shock syndrome	995.91	Sepsis
054.5	Herpetic septicemia	995.92	Severe sepsis
084.x	Malaria	999.31	Other and unspecified infection due to central venous catheter
098.89	Gonococcemia	
112.5	Disseminated candidiasis	999.32	Bloodstream infection due to central venous catheter
130.8	Multi-systemic disseminated toxoplasmosis	
		999.34	Acute infection following transfusion, infusion, or injection of blood and blood products
415.12	Septic pulmonary embolism	
449	Septic arterial embolism	
670.2	Major puerperal infection	999.39	Infection following other infusion, injection, transfusion, or vaccination
670.3	Puerperal septic thrombophlebitis	
**B. LOCALIZED INFECTIONS AND ASSOCIATED CONDITIONS**			
079.99	Unspecified viral infection	570	Acute and sub-acute necrosis of liver
276.2	Acidosis	573.9	Unspecified disorder of liver
286.x	Coagulation defects	593.9	Acute renal insufficiency
320.xx	Bacterial meningitis	681.xx	Cellulitis and abscess of finger and toe
321.x	Meningitis due to other organisms	682.x	Other cellulitis and abscess
322.9	Meningitis unspecified	728.86	Necrotizing fasciitis
421.x	Acute and sub-acute endocarditis	777.5x	Necrotizing enterocolitis in newborn
422.xx	Acute myocarditis	777.6	Perinatal intestinal perforation
480.x	Viral pneumonia	780.0	Alteration of consciousness
481	Pneumococcal pneumonia	780.2	Syncope and collapse
482.xx	Other bacterial pneumonia	780.4	Dizziness and giddiness
483	Pneumonia due to other unspecified organism	868.1x	Injury to intra-abdominal organs: with open wound into cavity
484.x	Pneumonia in infectious disease classified elsewhere	869.1	Internal injury to unspecified or ill-defined organs: with open wound into cavity
485	Bronchopneumonia, organism unspecified	996.6x	Infection and inflammatory reaction to internal prosthetic device, implant, graft
486	Pneumonia, unspecified	
557.0	Vascular insufficiency of the intestine	
567.0	Peritonitis and retroperitoneal infections	998.59	Other postoperative infection
567.1	Pneumococcal peritonitis	
567.2x	Other suppurative peritonitis	

#### Vital signs standardization group

The following data elements were obtained from the electronic triage vital signs for each ED patient: HR, RR, body temperature, and site of measurement, age, time between arrival and initial vital signs measurement, and reason for visit. HR’s outside the range of 30–300 beats/min, RR’s >120 breaths/min, and temperatures outside the range of 33–43°C (or 32–43°C if axillary) were rejected as spurious. Also rejected were HR’s and RR’s without a corresponding temperature measurement needed for calculation of temperature corrections of these rates.

### Outcomes

The primary outcomes for the Screening Tool Refinement group were the ROC values that describe the tool’s predictive ability relative to the “gold standard” identification of SS by physician chart review: sensitivity (and percentage of false negatives), specificity (and percentage of false positives), PPV, negative predictive value (NPV), and AUC. Given that the tool was designed to be incorporated into an automated, electronic screening tool that would run in the background for all patients entering the pediatric ED, our ROC test denominator consisted of all ED patient arrivals, since the possibility of a false negative or false positive result exists for all patients screened by the tool. Because ICCPS defined “sepsis” as the presence of infection accompanied by SIRS, and “severe sepsis” as sepsis accompanied by OD, the tool was designed to include both a SIRS and an OD component. We therefore added, as a secondary outcome, the percentage of cases that fired the SIRS component of the tool, which may be used to screen for the presence of sepsis in the absence of OD. Additionally, we included as a secondary outcome the median time from patient arrival to tool firing (in cases where firing occurred), which acts as a balancing measure for the purpose of weighting tool accuracy against the need for early identification of SS.

For the Vital Signs Standardization group, age-dependent (using the age intervals adopted by ICCPS) means and upper thresholds of normal (calculated as means plus a specified number of standard deviations) for HR and RR were determined. This methodology followed the original ICCPS criteria for upper limits of normal HR and RR, which were defined as the respective age-specific values of “mean + 2 SD.” Additionally, upper thresholds were calculated as “temperature corrected to 37°C” using both linear (22, 23) and exponential (24, 25) models for the temperature dependence of HR and RR. Linear temperature corrections were of the form: Corrected Rate (HR or RR) = Raw Rate − (C × ΔT), where C is a fixed number of beats/min or breaths/min, and ΔT = (Body Temperature −37.0°C). Exponential temperature corrections were of the form: Corrected Rate = Raw Rate ÷ Q_1_^ΔT^, where *Q*_1_ is the proportional increase in rate for a 1°C increase in temperature, and ΔT is as defined above.

### Analysis

#### Vital signs standardization

Analysis of the relationship of HR and RR with body temperature by age category led to the creation of exponential models that were used to derive a set of temperature corrected means and arbitrary upper thresholds of normal (mean + 2 SD, mean + 2.2 SD, etc.) HR and RR for each age category. Similar sets of corrected means and upper thresholds were also derived on the basis of temperature corrections suggested by previous studies. Ultimately, the choices of standards for abnormal HR and RR were based on each model’s plausibility and empirical ability to optimize the performance of the screening tool.

#### Screening tool refinement

Univariate analyses were performed for each measure incorporated into the screening tool to assess the association of abnormal values of that measure with gold standard identified SS. Additionally, the strengths of these associations at the time of the initial firing of the screening tool were compared with the respective associations looked at cumulatively throughout each patient’s hospital encounter. This allowed us to define abnormal values of particular metrics as “early” (i.e., commonly present at the initial firing of the tool) or “late” (i.e., not commonly present initially, but rather at some later time during the disease progression) indicators of SS. For both the early and late measures of association, the statistical significance of each association was determined using an exact chi-square test.

Refinement of the tool was accomplished through virtual PDSA cycle iterations, with the goal of successively improving ROC values with minimal increase in the mean interval between patient arrival and tool firing for Gold Standard SS cases. Using AUC as the measure of overall tool performance, the significance of our tool refinement process was evaluated using a chi-square test of the paired comparison between the original ICCPS (2) based tool and our final, revised tool (26).

To test whether the performance of our final tool was generalizable, we utilized a split-sample validation technique whereby the results from cases representing the Screening Tool Refinement group’s first month of patient arrivals were compared with cases representing the second month of arrivals. AUC was again selected as the measure of overall tool performance, and the difference in AUC for the two subsets was evaluated using an unpaired *t*-test (26).

## Results

### Characteristics of study subjects

#### Vital signs standardization group

A summary of the vital signs data for the Vital Signs Standardization group is shown in Table 2. Applying these standards to redefine tachycardia and tachypnea, using the ICCPS criteria of >2 SD above the mean for each age group, resulted in markedly higher thresholds than those published by ICCPS.

**Table 2:** **Characteristics of pediatric vital signs standardization group (*N* = 143,603)^a^**.

Age category	Heart rate			Respiratory rate			Body temperature^b^		
	*N*	Mean (beats/min)	SD	*N*	Mean (breaths/min)	SD	*N*	Mean (°C)	SD
Neonate^c^ (0–4 weeks)	2,681	153.3	19.3	2,626	42.2	9.3	2,709	37.1	0.5
Infant (>4 to <2 years)	45,418	141.8	23.0	44,707	32.1	9.3	46,121	37.5	1.0
Toddler and preschool (2 through 5 years)	43,240	121.4	22.7	42,384	24.4	6.8	43,826	37.2	0.8
School age child (6 through 12 years)	34,229	100.5	20.5	33,472	21.8	5.2	34,550	37.0	0.8
Adolescent and young adult (13 to <18 years)	16,216	88.2	18.9	15,949	19.5	4.5	16,397	36.8	0.6

*^a^This represents the number of cases with measurements of body temperature and either (or both) heart rate or respiratory rate*.

*^b^Temperatures collected via the axillary route were corrected by adding 0.2°C for neonates, and 0.6°C for all other age categories (18, 27)*.

*^c^Combines the “newborn” and “neonate” categories as defined by ICCPS (2)*.

#### Screening tool refinement group

Some general characteristics of the Screening Tool Refinement group are listed in Table 3. Notably, inspection of Gold Standard SS cases identified by physician chart review revealed that <20% of all SS cases were actually identified as such by discharge coding, with the remainder being “under-coded” with a less severe diagnosis. Moreover, over 20% of these SS cases had negative culture results for infectious organisms (bacteria, viruses, or fungi) in blood, CSF, or urine, and about 10% of the cases had negative respiratory culture results as well. Additionally, while overall mortality was quite low (8/7,402, or 0.1%) among the children who visited the ED during the 2-month study period, nevertheless 38% of these deaths (three out of eight) occurred among the children identified as Gold Standard SS cases. In a large majority of Gold Standard SS cases, the patient did not arrive at the ED in a condition of SS but rather progressed to that condition during the course of the hospital stay. This is consistent with our finding that the median time from patient arrival to initial tool firing for these cases was 11.1 h, with the 25th and 75th percentiles firing at 5.0 and 22.2 h, respectively.

**Table 3:** **Characteristics of screening tool refinement group (*N* = 7,402)**.

Characteristics	Mean (SD) or % (*N*)
Age category	5.9 years (5.5)
Neonate^a^ (0–4 weeks)	2.5% (184)
Infant (>4 weeks to <2 years)	33.1% (2,452)
Toddler and preschool (2 through 5 years)	25.3% (1,873)
School age child (6 through 12 years)	22.2% (1,640)
Adolescent and young adult (13 to <18 years)	16.9% (1,253)
Gender
Male	54.8% (4,056)
Female	45.2% (3,346)
Admitted as inpatient	21.1% (1,561)
Coded as severe sepsis or septic shock at discharge	0.1% (7)
Total severe sepsis/shock (by physician review)	0.5% (38)
Among age groups: neonate^a^ (*N* = 184)	1.6% (3)
Infant (*N* = 2,452)	0.6% (15)
Toddler and preschool (*N* = 1,873)	0.4% (7)
School age child (*N* = 1,640)	0.3% (5)
Adolescent and young adult (*N* = 1,253)	0.6% (8)
Severe sepsis/shock cases with negative blood, CSF, and urine (B/C/U) cultures	21.2% (8/38)
Severe sepsis/shock cases with negative B/C/U and respiratory cultures	10.5% (4/38)
Median length of stay among severe sepsis/shock cases (days)	14.5 (Range: 0.7–78.7)
Overall mortality (all causes)	0.1% (8)
Mortality among severe sepsis/shock cases (*N* = 38)	7.9% (3)

*^a^Combines the “newborn” and “neonate” categories as defined by ICCPS (2)*.

### Main results

#### Vital signs standardization

**Figure 2:**
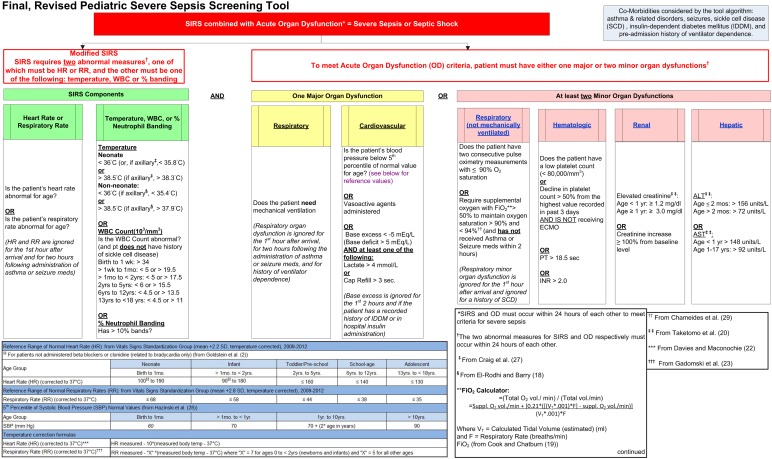
**Final, revised pediatric severe sepsis screening tool**.

Based on their empirical utility in identifying Gold Standard SS cases, the age-specific thresholds for tachycardia and tachypnea were selected using models employing a linear temperature correction to derive standardized HR’s and RR’s corrected to 37°C. For HR, the model assumed a fixed increase of 10 bpm/°C of temperature increase, as proposed by Davies and Maconochie (22). Similarly, for RR the model used a fixed increase of 7 breaths/min °C for neonates (which also includes the original ICCPS category “newborn”) and infants; and 5 breaths/min °C for all other age groups, as proposed by Gadomski et al. (23). The final optimized temperature corrected thresholds, representing mean HR +2.2 SD and mean RR +2.8 SD for each age category, are shown in Figure 2 (2, 18–20, 22, 23, 27–29).

#### Screening tool refinement

##### Univariate associations of SIRS and OD metrics with gold standard SS

The results of univariate analyses of the associations of individual SIRS and OD metrics with physician identified Gold Standard SS cases are shown in Table 4. Among SIRS measures [HR, RR, temperature, white blood cell (WBC) count, and neutrophil percent banding], the strongest associations – defined as the ratio (relative risk) of the incidence of cases with ≥1 abnormal value of that measure among Gold Standard SS cases to that among non-Gold Standard SS cases – were found for temperature corrected HR and RR, with ratios of 13.8 and 9.6, respectively. This finding led us to redefine the tool’s criteria for a positive finding of SIRS from the original ICCPS definition (which required abnormal values for two SIRS components, one of which must be temperature, WBC count, or neutrophil percent banding) to a more restrictive definition that additionally requires the second abnormal SIRS component to be either HR or RR. In terms of tool performance, this redefinition allowed us to markedly improve overall specificity without any loss in sensitivity.

**Table 4:** **Association of severe sepsis with abnormal SIRS and OD metrics**.

Measure	Association based on cases having ≥1 abnormal value					Association based on cases having ≥1 abnormal value				
	at time of initial tool firing					at any time during hospital encounter				
	% Of gold	Relative	Total	*p*	Early	% Of gold	Relative	Total	*p*	Late
	standard sepsis	risk of ≥1	number of		indicator	standard sepsis	risk of ≥1	number of		indicator
	cases with	abnormal	patients		of severe	cases with	abnormal	patients		of severe
	≥1 abnormal	value^a^	measured		sepsis^b^	≥1 abnormal	value^a^	measured		sepsis^c^
	value		or tested			value		or tested		
Temp. *or* WBC count *or* % banding	100	4.0	7, 377	< 0.0001	✓	100	4.0	7, 377	< 0.0001	
Temp. corrected HR *or* RR	97.4	9.7	7, 392	< 0.0001	✓	97.4	9.7	7, 392	< 0.0001	
Temp. corrected HR	81.6	13.8	7, 392	< 0.0001	✓	84.2	14.2	7, 392	< 0.0001	
Temperature	67.6	3.4	7, 369	< 0.0001	✓	84.2	4.3	7, 370	< 0.0001	
Temp. corrected RR	60.5	9.6	7, 390	< 0.0001	✓	89.5	14.0	7, 390	< 0.0001	
Neutrophil % banding	59.3	3.2	358	< 0.0001	✓	61.3	3.2	364	< 0.0001	
Use of mechanical ventilation	57.9	137.5	7, 402	< 0.0001	✓	68.4	157.5	7, 402	< 0.0001	
White blood cell (WBC) count	55.6	1.8	2, 033	0.003	✓	78.4	2.5	2, 036	< 0.0001	
Prothrombin time (PT)/INR	50.0	25.9	273	< 0.0001	✓–	47.1	24.4	276	< 0.0001	
ALT or AST level	42.3	6.4	1, 147	< 0.0001	✓–	45.7	6.9	1, 157	< 0.0001	
Systolic blood pressure	42.1	13.6	7, 337	< 0.0001	✓–	65.8	21.0	7, 337	< 0.0001	✓
CNS function	39.4	10.4	7, 326	< 0.0001		60.5	15.9	7, 332	< 0.0001	✓
Peripheral pulse strength (weak)	34.3	73.1	6, 857	< 0.0001		50.0	106.6	6, 860	< 0.0001	✓–
Lactate	33.3	2.5	65	0.19		19.0	1.4	74	0.50	
Base deficit	32.0	2.5	285	0.02		48.5	3.9	296	< 0.0001	✓–
Lactate + base deficit	27.3	2.8	52	0.15		19.0	2.0	62	0.43	
Capillary refill time	25.7	13.0	7, 139	< 0.0001		47.4	24.0	7, 142	< 0.0001	✓–
Use of supplemental O_2_ w/o vent	23.7	12.9	7, 402	< 0.0001		55.3	29.3	7, 402	< 0.0001	✓
Use of vasoactive agents	13.2	242.2	7, 402	< 0.0001		31.6	465.1	7, 402	< 0.0001	
Creatinine	11.8	31.6	1, 917	< 0.0001		10.8	29.1	1, 922	< 0.0001	
Platelet count	8.8	7.9	2, 012	0.008		24.3	21.0	2, 018	< 0.0001	
Bilirubin	3.8	2.3	1, 118	0.36		8.6	5.2	1, 128	0.02	

*^a^ Calculated as the ratio of the incidence of cases with ≥1 abnormal value of the measure among gold standard severe sepsis (GSSS) cases to the incidence among non-GSSS cases*.

^b^ Measures for which >50% of gold standard severe sepsis cases exhibit abnormal values at the time of initial tool firing are denoted as stronger early indicators of severe sepsis with the symbol “✓.” Measures for which 40–50% of gold standard severe sepsis cases exhibit abnormal values at the time of initial tool firing are denoted as weaker early indicators of severe sepsis with the symbol “✓–.”

^c^ Measures for which <50% of gold standard severe sepsis cases exhibit abnormal values at the time of initial tool firing and >50% of gold standard severe sepsis cases exhibit abnormal values at any time during the hospital encounter are denoted as stronger late indicators of severe sepsis with the symbol “✓.” Measures for which <40% of gold standard severe sepsis cases exhibit abnormal values at the time of initial tool firing and 40–50% of gold standard severe sepsis cases exhibit abnormal values at any time during the hospital encounter are denoted as weaker late indicators of severe sepsis with the symbol “✓–.”

The univariate analyses also allowed us to identify certain SIRS and OD component measures as “early” indicators of SS (i.e., exhibiting abnormal values in a majority or near-majority of gold standard SS cases at the initial firing of the screening tool), or as “late” indicators (i.e., exhibiting abnormal values in a minority of Gold Standard SS cases at the time of initial tool firing, but in a majority or near-majority of Gold Standard SS cases at some later time during the patients’ hospital encounters). These findings are summarized in Table 4.

Several measures incorporated into the original ICCPS based tool were found to have a very low incidence among Gold Standard SS cases or to have an association that was not statistically significant. Specifically, a very small percentage of Gold Standard SS cases showed abnormal bilirubin (3.8% at initial tool firing, *p* = 0.36; 8.6% at any time during the patient encounter, *p* = 0.02), and the association of abnormal lactate – either alone or in combination with abnormal base deficit – with Gold Standard SS was not statistically significant (lactate alone: *p* = 0.19 at initial tool firing, and *p* = 0.50 cumulatively during the encounter; lactate + base deficit: *p* = 0.15 initially, and *p* = 0.43 cumulatively). However, the absence of an association of Gold Standard SS with lactate may reflect the small number of instances that lactate tests were ordered (*N* = 65 initially, and *N* = 74 cumulatively) for patients in the Screening Tool Refinement group.

#### Tool refinement process

A summary of the changes to the original ICCPS criteria leading to our final pediatric SS screening tool is given in Table 5. These changes were generally classifiable into three categories: (1) the addition of new criteria to improve tool sensitivity; (2) the removal or modification of criteria to improve tool specificity; and (3) the use of patient history (triage) information or medication administration data to identify classes of conditions – such as asthma, seizures, diabetic ketoacidosis, and sickle-cell disease (SCD) – that were likely to cause false positive tool firing, and to suppress the firing of portions of the tool for these patients.

**Table 5:** **Alterations/revisions made to the original ICCPS (2) version of the pediatric severe sepsis tool**.

**Respiratory organ dysfunction (OD):** cases not requiring mechanical ventilation but meeting the PEARS 2012 criteria (29) for respiratory dysfunction (FiO_2_ >50% with oxygen saturation >90 and <94%), or having two successive oxygen saturations ≤90%, were redefined as minor respiratory OD and required a second minor OD to trigger the full OD component of the tool. FiO_2_ was estimated using EMR entries of O_2_ flow rate and an estimate of tidal volume based on age, weight, and gender (19)
**Axillary temperatures:** a correction of +0.6°C for non-neonates (18), and +0.2°C for neonates (27), was added to temperature measurements made via the axillary route
**Asthma and seizures:** abnormal values for HR, RR, and respiratory OD were suppressed for 2 h following administration of asthma medications [albuterol, dexamethasone, epinephrine (intramuscular or subcutaneous), methylprednisolone, magnesium sulfate, or terbutaline] or seizure medications (lorazepam, levetiracetam, fosphenytoin, or phenobarbital)
**First hour HRs, RRs, and respiratory OD:** tool specificity was improved by suppressing abnormal values for HR and RR, and the respiratory OD component of the tool, for the first hour after patient arrival. This also allowed for the identification and treatment of asthmatic and seizure patients. Importantly, tool sensitivity and median time from patient arrival to detection of severe sepsis were unaffected
**CNS OD** was dropped from the tool, improving specificity without affecting sensitivity
**Abnormal prothrombin time (PT)** was added as an additional indicator of hematologic OD alongside INR, resulting in improved tool sensitivity
**Bilirubin** was dropped as a measure of hepatic OD due to its very weak association with pediatric severe sepsis
**Peripheral pulse strength** was dropped as a “Minor” measure of cardiovascular OD, improving specificity without affecting sensitivity
**Data entry error** detection algorithms were added to drop unreasonable values for vital signs, systolic blood pressures (SBP’s) that represented extreme drops from their previous readings, and vital signs with two or more disparate values having identical recorded date and time values. This was necessary to overcome difficulties inherent in the application of the tool to systems that utilize electronic medical records
**Sickle-cell disease (SCD):** abnormal WBC count values and minor respiratory OD triggers were suppressed for patients with an entered history of SCD, improving tool specificity. The suppression of abnormal WBC count for this group followed from finding that roughly 50% of SCD patients exhibited abnormally high WBC counts in the absence of severe sepsis
**Diabetes:** abnormal base deficit readings were suppressed for *all* patients for the first 2 h after arrival and for patients administered insulin or with an entered history of diabetes. This allowed for the identification and treatment of patients with diabetic ketoacidosis
**Abnormal AST** level was added as an additional indicator of Hepatic OD (the original tool considered only ALT), resulting in improved sensitivity
**Ventilator dependence**, as identified and entered at triage, resulted in a suppression of the respiratory OD trigger for these patients, improving tool specificity
**“SBP2”:** the PALS criteria for systolic hypotension (28) were substituted for those given in the ICCPS 2005 supplement (16). This increased the specificity of the tool modestly from versions employing the latter criteria, and greatly from versions employing the SBP criteria given in the original ICCPS 2005 guidelines (2)
**Lactate** was restricted as a “Minor” indicator of cardiovascular OD to cases where elevated lactate was accompanied by abnormal base deficit, i.e., acidosis
**Redefinition of abnormal HR and RR:** the set of empirically derived cut-offs defining tachycardia and tachypnea, derived from our Pediatric Vital Signs Standardization Group, were substituted for those given in the original ICCPS guidelines
**“SIRS2”:** the criteria for triggering the SIRS component of the tool were redefined to require one abnormal value of either temperature, WBC, or neutrophil banding; and one abnormal value of either HR or RR
**Temperature corrections of HR and RR** were added, based on the linear relationships reported by Davies and Maconochie (22), and Gadomski et al. (23), respectively. Together with the redefinition of abnormal HR and RR, and the redefinition of criteria for SIRS, this resulted in a substantial increase in tool specificity with no loss of sensitivity

**Figure 3:**
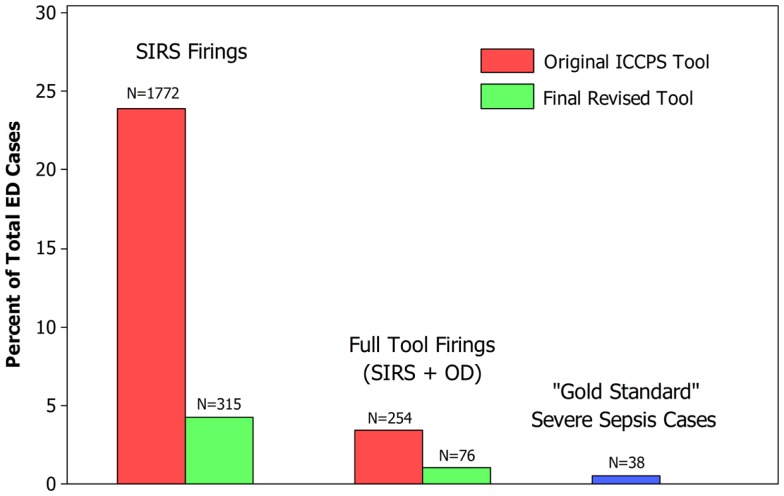
**Comparison of ICCPS (2) based tool with final revised tool**.

The criteria incorporated in the final revised version of our tool are shown in Figure 2. A comparison of the previously described primary and secondary outcome measures for the initial ICCPS and final revised screening tools is given in Table 6, showing that the PPV increased from 14.6% in the original tool to 48.7% in the final tool – a more than threefold improvement – while maintaining the same high sensitivity (97.4%) in both versions. This improvement was highly significant (*p* < 0.0001) in terms of the paired comparison of AUC between the two versions of the tool. Simultaneously, the percentage of children meeting SIRS criteria was reduced from 23.9 to 4.3%, a nearly sixfold improvement. Because all firings of the full tool (SIRS + OD) also represent SIRS firings, this decrease in SIRS firings in the absence of any decrease in full tool sensitivity implies that these additional firings in the original version were false positives from the perspective of a SS screening tool. The overall reduction of false positive firings for both the SIRS component of the tool and the overall SS tool (SIRS + OD) is illustrated in Figure 3.

**Table 6:** **Comparison of initial and final versions of the pediatric severe sepsis screening tool**.

Measure	Original ICCPS (2) based tool	Final revised tool
Total cases/gold standard severe sepsis (GSSS) cases	7,402/38	7,402/38
SIRS firings^a^/percentage of total cases	1,772/23.9	315/4.3
False positives/false negatives	217/1	39/1
Sensitivity/specificity (%)	97.4/97.1	97.4/99.5
Positive predictive/negative predictive value (%)	14.6/99.99	48.7/99.99
Area under the curve (AUC)	0.972	0.984
Median time from arrival to tool firing for GSSS cases	10.3 h (P25 = 2.8, P75 = 20.6)	11.1 h (P25 = 5.0, P75 = 22.2)

*^a^SIRS Firing indicates that the tool “detected” the presence of SIRS based on the criteria used by the tool*.

#### Validation

The split-sample validation analysis tested the null hypothesis that the AUC for the results from the first month of Screening Tool Refinement group patient data did not differ from that for the second month. The relevant ROC outcomes for the two data subsets were as follows: month (#1) *N* = 3,713, sensitivity = 96.0%, specificity = 99.5%, AUC = 0.9774, standard error (SE) = 0.02; month (#2) *N* = 3,689, sensitivity = 100%, specificity = 99.5%, AUC = 0.9973, and SE = 0.0006. The resultant AUC difference of 0.0199 was not statistically significant (*p* = 0.32), thus confirming that the tool outcomes were generalizable over the two independent study sub-samples.

## Limitations

The Screening Tool Refinement group consisted of 7,402 cases, representing 2 months of ED visits at a single children’s hospital. Among this group, 38 cases of Gold Standard SS were identified. A larger study population drawn from multiple children’s hospitals and with more Gold Standard SS cases would be desirable. However, a principal strength of our methodology lay in its establishment of a “gold standard” identification of SS cases based on physician chart review of 480 patient cases that was consistent across the study group and independent of the appraisal of the screening tool. While it may be argued that a true gold standard determination should involve chart reviews of all 7,402 cases, such a procedure is not practical, especially given that the next step in tool validation would involve the expansion of the study to a much larger patient population. In an attempt to review all cases having some likelihood to be instances of SS, we employed a very wide diagnostic “net” that included all cases that fired the tool, all cases with a diagnosis of any disseminated infection (including but not limited to septic shock, SS, and sepsis), additional cases with a coded localized infection that has been known to progress to sepsis, and even cases with coded symptoms of organ dysfunctions (including hepatic, renal, CNS, and metabolic) likely to be present in patients with SS. We believe it very unlikely that a case of SS would escape inclusion by this net, and we note that other published validations of adult sepsis tools, such as that of Moore et al. (15), have employed a much narrower net. The latter study, for example, selected only cases that fired the tool or had a coded diagnosis of a disseminated infection for inclusion in the chart review process to determine gold standard sepsis status.

As stated earlier, our objective was to create an improved tool for detection of SS in terms of specificity, PPV, and median time from patient arrival to SS detection. We recognize that the attempt to create an “optimized” tool involves a trade-off; for example, it was possible to create a tool with a shorter median time to sepsis detection at the cost of a considerable decrease in specificity. Our initial SS screening tool based on the original ICCPS criteria proved to be quite sensitive but not very specific, which would likely lead to trigger fatigue in an ED setting. Our approach was to prioritize high specificity while minimizing (but not eliminating) an inevitable increase in median time to sepsis detection. Thus, for example, our final tool suppresses firing based on abnormal HR, RR, and respiratory organ dysfunction for the first hour following patient arrival in order to allow entry of additional information that may identify such patients as having non-septic conditions such as asthma, seizure, or SCD. This suppression of firing resulted in an increase in the first quartile time to sepsis detection by the tool among gold standard cases by 1.7 h (from 3.3 h without suppression to 5.0 h with suppression). However, it also decreased the number SIRS firings among non-Gold Standard SS cases by 26%, from 377 to 278 over the 2-month study period, without decreasing the sensitivity of the tool in identifying cases of SS.

Other significant limitations resulted from the retrospective nature of our study; consequently, the scope of our data capture was restricted to patient information and tests taken or ordered at a time before a specific sepsis protocol was in place at the Screening Tool Refinement study hospital. In particular, our ability to assess the association between Gold Standard SS and lactate or neutrophil percent banding was limited by the infrequent ordering of these tests. Similarly, our ability to determine the association of Gold Standard SS with peripheral pulse strength or capillary refill time was confounded by the documentation practice of assigning default values of “Normal” to these measures unless specifically noted otherwise, which removed the ability to distinguish between cases where no actual measurement was taken (i.e., those with missing values) and cases where a measurement was taken and found to be normal.

Additionally, we were unable to assess CNS function among the Screening Tool Refinement group using the preferred ICCPS measure, the Glasgow Coma Score, because that measure was seldom utilized at the study hospital except for trauma cases. We therefore designed an alternative measure of CNS function based on the Nursing Neurological Assessment in the Cerner EMR, which was recorded at triage and completed for nearly all patients in the Screening Tool Refinement group. While this available EMR-based assessment represented the best source of CNS function appraisal available for our study group, our finding that CNS function measured in this manner is not a strong early indicator of Gold Standard SS should not be generalized so as to discourage the development of alternative measures of neurologic function that may show a stronger association with Gold Standard SS.

## Discussion

The ICCPS guidelines represented a consensus derived from existing definitions and criteria for SS in adults that were extended to pediatrics. Because its guidelines were the result of consensus, ICCPS did not empirically derive screening tool criteria for SS that optimized sensitivity, specificity, PPV, and time from patient arrival to sepsis detection. With respect to vital signs, the tables found in the original ICCPS document were based on data from normal, healthy children obtained for purposes not related to the optimal detection of SS.

Our methodology combined a retrospective, iterative method of screening tool optimization, an empirically derived set of age-specific thresholds for abnormal HR and RR, and an intensive physician chart review process to establish a “gold standard” identification of SS. This resulted in a pediatric SS screening tool with a PPV that is significantly higher than any previously published. Furthermore, the high predictive value of our tool extended to cases that did not show positive blood, CSF, urine, or respiratory culture results – either for bacteria, viruses, or fungi – and so might have escaped early detection by traditional methods that rely on such positive culture results. The use of antibiotics prior to the time when the cultures were obtained may have hindered bacteria from growing in the various samples. Additionally, rapid viral antigen tests are not inclusive of the many types of viruses that may infect patients.

In addition to our tool refinement methodology and our derivation of an improved set of age-specific vital signs thresholds, our study resulted in several other improvements to the original ICCPS components and criteria:

(1) Analysis and classification of ICCPS defined component measures of SIRS or OD as “early” or “late” indicators of SS according to the association of abnormal values for each of these measures with Gold Standard SS and to the time during the hospital encounter with respect to initial tool firing when these abnormal values occurred;
(2) Redefinition of SIRS in a way that ensures that those SIRS components having the greatest association with Gold Standard SS – namely, temperature corrected values of HR or RR – become mandatory criteria for the screening tool to “detect” the presence of SIRS; and
(3) Consolidation of the ICCPS defined age categories “Newborn” and “Neonate,” which no longer require separate sets of thresholds (with the sole exception of those for abnormal WBC Count).

While we believe that our screening tool represents a significant advancement in the prediction of SS in children, we also recognize the need for continued work toward improved accuracy and timeliness in its detection. One avenue for future investigation lies in the reexamination of pediatric age categories, particularly those chosen for vital signs thresholds. Given that a major challenge in designing a SS tool for children is the age-specific nature of what is “abnormal” for a given vital sign or laboratory result, we believe that an optimal pediatric sepsis screening tool should be based on empirically derived age categories of reasonable uniformity in terms of thresholds for “abnormal” obtained from children presenting to healthcare providers.

There is also a need for sepsis detection tools that alert clinicians to the likelihood of sepsis at an early point during a patient encounter, even as early as the time of ED triage. Obviously, the development of such a triage sepsis screening tool presents a unique challenge: the need to provide a reasonably sensitive and specific prediction of sepsis in the absence of laboratory results, and so based disproportionately on readily observed clinical signs, vital signs, and patient history. With the goal of improving the detection and treatment of pediatric sepsis – where each hour of delay increases the risk of death – it remains a priority to continue our efforts to detect this disease more quickly, accurately, and efficiently.

## Conclusion

Our iterative, virtual PDSA cycle method of tool refinement, empirically derived thresholds of abnormal HR and RR in an ED setting, and independent, physician review-based identification of “gold standard” SS cases resulted in a pediatric SS screening tool for the ED having improved predictive value compared with that of a tool derived from the original ICCPS (2) criteria.

Additionally, our analysis of component SIRS and OD metrics led to the identification of strong “early” indicators of SS. In descending strength of association with Gold Standard SS, these were: temperature corrected HR, temperature, temperature corrected RR, neutrophil percent banding, need for mechanical ventilation, and white blood count. Other SIRS and OD component metrics, such as systolic blood pressure (SBP) and CNS function, were also found to have a strong association with SS but were identified as “late” indicators and so relatively less useful in designing a tool whose goal is the timely detection of SS.

## Author Contributions

Robert J. Sepanski and Sandip A. Godambe contributed to all aspects of this study, which occurred at two sites (Memphis and Norfolk). Robert J. Sepanski drafted the manuscript, and Sandip A. Godambe, Christine S. Bovat, and Arno L. Zaritsky critically revised the manuscript. Christine S. Bovat and Arno L. Zaritsky were involved in the project’s conception, design, IRB and grant applications, and data acquisition, analysis and interpretation at the Norfolk site and also assisted with data interpretation from the other site. Christopher D. Mangum was involved in the project’s conception, design, data acquisition, and interpretation at the Memphis site and review of the manuscript. Samir H. Shah was involved in the conception, design, and IRB application of the Memphis phase of the project, and in review of the final manuscript.

## Conflict of Interest Statement

The authors declare that they have no competing interests. This work was partly funded by an institutional Children’s Foundation Grant (Norfolk, VA, USA). This grant has no requirements that may bias this work. No author will gain or lose financially from the publication of this manuscript including the Children’s Foundation. No patents are being held or pursued relating to the content of this manuscript.

## Abbreviations

AUC, area under the curve; ECMO, extracorporeal membrane oxygenation; ED, emergency department; EMR, electronic medical record; GSSS, “gold standard” severe sepsis (or septic shock); HR, heart rate; ICCPS, international consensus conference on pediatric sepsis; NPV, negative predictive value; OD, acute organ dysfunction; P25, 25th percentile (or first quartile); P75, 75th percentile (or third quartile); PDSA, plan-do-study-act; PPV, positive predictive value; ROC, receiver operating characteristics; RR, respiratory rate; SAS^®^, statistical analysis system (SAS Institute Inc., Cary, NC, USA); SBP, systolic blood pressure; SCD, sickle cell disease; SE, standard error; SIRS, systemic inflammatory response syndrome; SS, severe sepsis.
